# The Role of Templating in the Emergence of RNA from the Prebiotic Chemical Mixture

**DOI:** 10.3390/life7040041

**Published:** 2017-10-31

**Authors:** Andrew S. Tupper, Kevin Shi, Paul G. Higgs

**Affiliations:** 1 Origins Institute and Department of Biochemistry and Biomedical Science, McMaster University, Hamilton, ON L8S 4L8, Canada; tuppea2@mcmaster.ca; 2 Origins Institute and Department of Physics and Astronomy, McMaster University, Hamilton, ON L8S 4K1, Canada; shik6@mcmaster.ca

**Keywords:** RNA world, non-enzymatic template-directed replication, chirality, regioselectivity, prebiotic chemistry, symmetry breaking

## Abstract

Biological RNA is a uniform polymer in three senses: it uses nucleotides of a single chirality; it uses only ribose sugars and four nucleobases rather than a mixture of other sugars and bases; and it uses only 3′-5′ bonds rather than a mixture of different bond types. We suppose that prebiotic chemistry would generate a diverse mixture of potential monomers, and that random polymerization would generate non-uniform strands of mixed chirality, monomer composition, and bond type. We ask what factors lead to the emergence of RNA from this mixture. We show that template-directed replication can lead to the emergence of all the uniform properties of RNA by the same mechanism. We study a computational model in which nucleotides react via polymerization, hydrolysis, and template-directed ligation. Uniform strands act as templates for ligation of shorter oligomers of the same type, whereas mixed strands do not act as templates. The three uniform properties emerge naturally when the ligation rate is high. If there is an exact symmetry, as with the chase of chirality, the uniform property arises via a symmetry-breaking phase transition. If there is no exact symmetry, as with monomer selection and backbone regioselectivity, the uniform property emerges gradually as the rate of template-directed ligation is increased.

## 1. Introduction

An important feature of life is that it makes frequent use of a well-defined set of molecules, but it does not use many other kinds of molecules that have rather similar chemical properties. This has been called the ‘lego principle’ [1], and it has been proposed that this feature could be used as a signature of life on other planets. This principle applies to amino acids, where the set of possible amino acids is much larger than the 20 used in biological proteins [2], and to nucleotides, where there is a huge diversity of sugars [3] and nucleobases [4,5] that could potentially form polymers similar to RNA and DNA. Non-living chemistry is governed by thermodynamics and reaction kinetics. Similar molecules will have similar free energies of formation and will undergo similar reactions; hence, they will be produced in similar quantities. Living biochemistry is autocatalytic. A subset of molecules is able to catalyze formation of more of the same set of molecules that it requires for growth and reproduction. Because there is continued flow of energy and matter into a living system, the relative frequencies of different molecules can be maintained far from what would be found in thermodynamic equilibrium.

One example of the way autocatalysis maintains biased sets of molecules is the homochirality observed in nucleic acids and proteins [6]. Theoretical models assume that the formation of molecules of a given enantiomer (*D* or *L*) is catalyzed by molecules of the same handedness. In the simplest model of Frank [7], monomers are autocatalytic; however, more complex models have been studied in which the catalysts are dimers [8] or polymers [9,10]. All these models share the feature that it is the monomer synthesis reaction that is catalyzed (i.e., the synthesis of ribose or ribonucleotides, if we apply this to RNA). Here we consider the alternative that the asymmetric autocatalysis comes from template-directed synthesis of complementary oligomers, rather than from the catalysis of nucleotide synthesis. We assume that oligomers of uniform chirality (either *D* or *L*) are efficient templates for the ligation of shorter oligomers of the same chirality, whereas oligomers of mixed chirality are unable to act as templates. We show that if the template-directed reaction rate is fast, a symmetry-breaking phase transition occurs in which one or other enantiomer dominates the system.

Biological nucleic acids show two other kinds of uniform properties in addition to chirality. Firstly, biology uses a uniform set of monomers, rather than a mixture of many other similar molecules with different sugars, different bases, or both. Only four nucleotides are used in genetic information storage and transcription in DNA and RNA (although many modified nucleotides are used in specific positions in structural RNAs, such as tRNAs). We refer to the question of why these particular nucleotides are used as the monomer selection problem. Secondly, biology uses regular 3′-5′ bonds between ribose sugars rather than a mixture of 3′-5′ and 2′-5′ bonds. We refer to this as the backbone regioselectivity problem. The central point of this paper is that the monomer selection and backbone regioselectivity problems are similar problems to the chirality problem, and we may use a similar theory to explain all three. Our theory depends on two propositions: (1) that uniform oligomers of one kind are templates that catalyze synthesis of further oligomers of the same kind (i.e., the same chirality, the same monomers, or the same bond type); and (2) that uniform oligomers are good templates, but mixed oligomers (i.e., mixed chirality, mixed monomers, or mixed bond types) are not. These two propositions are supported by experiment in several ways, as we will now discuss.

In the case of chirality, Bolli et al., [11] studied template-directed ligation of tetramers of pyranosyl-RNA, and showed that the ligation of the homochiral tetramers is faster by at least two orders of magnitude than the ligation of tetramers in which one of the nucleotides has the opposite chirality. In this example, both propositions are clearly satisfied. With standard RNA, Joyce et al. [12] studied the polymerization of G monomers using a poly(C) template of the *D* enantiomer, and showed that template-directed synthesis of G oligomers is efficient when the G monomers are also of the *D* enantiomer, and not when they are of the *L* enantiomer. When a racemic mixture of *D*/*L* monomers was used, the *L* monomers inhibited the growth of the G oligomers to some extent, but the template-directed reaction was still an improvement over the case with no template at all. The chiral inhibition effect was presented as a problem for theories of the origin of life in [12], however we see this effect as part of the solution, in the sense that some kind of chiral inhibition is necessary to drive the symmetry breaking between *D* and *L*. Without this, we would expect the two systems to mix and coexist.

The two propositions also apply with regard to the monomer selection problem. Oligomer duplexes of RNA and DNA have been studied in many combinations [13]. It is found that duplexes of pure RNA or pure DNA have higher melting temperatures than hybrid duplexes (in which one strand is pure RNA and one is pure DNA) and mixed duplexes (in which strands are mixtures of ribonucleotides and deoxyribonucleotides). RNA and DNA are rather similar in structure, and hybridization between the two is clearly possible. Hybridization between the two allows for transcription in today’s organisms, and is also essential for the transfer of information from RNA to DNA that is proposed to occur at the end of the RNA World. Scenarios have also been proposed in which DNA arose concurrently with RNA, rather than as a late successor [13]. However, the essential point here is that, even with two very similar kinds of monomers, each has a preference for its own kind, and this will tend to drive separation between the two, as we will see in the theoretical models below. 

Several other alternative nucleic acid-like polymers have been studied that differ more substantially in structure from RNA. Melting temperatures of duplexes of these polymers differ significantly, and may be either higher or lower than for RNA duplexes [14]. This suggests that RNA may be optimized for the conditions in which replication was originally occurring [15,16]. Some alternative nucleic acids can form hybrid duplexes with RNA, and some cannot. For example, Schöning et al. [17] studied four pairs of complementary oligomers, each made with TNA (α-threofuranosyl nucleic acid), RNA, and DNA backbones, and measured the melting temperatures of the 24 possible hybrid duplexes. They found a general tendency to favour uniform duplexes, although the results are complex, and we will return to them in the discussion section. 

Monomer selection also involves a choice between nucleotides that differ in bases but have the same backbone. It is clear that strands made from the standard ACGU ribonucleotide alphabet are effective as templates for strands of the same alphabet. Non-enzymatic, template-directed synthesis of RNA and DNA has been studied [18], particularly with regard to the fidelity of sequence replication. A good monomer alphabet for genetics should allow little mismatch pairing. The possibility of GU pairing in the ACGU alphabet of RNA, and the absence of this possibility in the ACGT alphabet of DNA, is a potential reason for the transfer of information from RNA to DNA during evolutionary history [18]. Furthermore, a number of other base pairs have been studied that can be used as an extension of the coding repertoire of RNA and DNA [19,20]. These bases are ‘orthogonal’ to the standard bases, i.e., there should be little or no pairing between them and the standard bases. This means that sequences made of either the standard alphabet or the alternative alphabet would be templates for complementary sequences in the same alphabet, which is our proposition 1. However alternative pairs that are compatible with the RNA or DNA helix may not satisfy proposition 2, because the mixed sequences may still be good templates in this case. Studies of template-directed synthesis [21] have shown that the rate of nucleotide addition after a mismatch is much lower than after a match. This illustrates a close parallel between the monomer selection problem and the chirality problem, where the addition of a monomer of the wrong chirality can slow down the growth of an oligomer [12]. Although these effects cause slower average growth of oligomers, they actually increase the degree of uniformity of the longer oligomers. Mismatch inhibition increases the overall fidelity of RNA replication [21], and we expect that chiral inhibition would increase the enantiomeric excess of longer oligomers for the same reason.

Early studies on oligomerization of activated guanosine nucleotides using poly(C) templates [22,23] found that G-G bond formation is regioselective with a majority of 3′-5′ bonds, i.e., the template favours formation of the same bond type in the complementary strand (proposition 1 above). It was additionally shown [24] that the A structure of the nucleic acid helix is important in bringing the nucleotides together in the correct conformation for 3′-5′ bond formation. Furthermore, there have been several experimental studies that look at the effects of mixing 2′-5′ and 3′-5′ bonds. When regular 3′-5′ strands are used as templates, the template-directed synthesis of the complementary strand is regioselective for 3′-5′ bonds [25]. The duplex melting temperature is found to decrease systematically by around 15 °C as 2′-5′ bonds are added to the regular 3′-5′ structure [26,27]. This also suggests that strands of uniform bond type should be better templates than mixed strands (proposition 2). If 2′-5′ bonds and 3′-5′ bonds are similar with respect to rates of formation and hydrolysis, then the possibility arises that that strands of uniform bond type can be selected by symmetry breaking, as we discuss in this paper. However, it is already known that 2′-5′ bonds may be much less stable to hydrolysis than 3′-5′ bonds [28], hence, there is no true symmetry in this problem, and it is possible for one bond type to be more frequent than the other without there being a symmetry-breaking transition. A recent experimental study has shown that iterative degradation and repair of bonds gradually converts 2′-5′ bonds to 3′-5′ bonds [29] because bond formation in the context of the existing helix is regioselective. This effect is included in the model of backbone regioselectivity given here.

The aim of this paper is to present a simple computational model that is able to treat the problems of chirality, monomer selection, and backbone regioselectivity in the same way. We propose that all three types of order emerge when the rate of non-enzymatic template-directed synthesis is high, due to the fact that uniform strands are templates for their own kind, and that uniform strands are better templates than mixed strands. This mechanism is likely to occur at the level of oligomers, and does not require the synthesis of strands that are long enough to function as specific ribozymes.

## 2. Materials and Methods

### 2.1. Reaction Schemes

**Figure 1:**
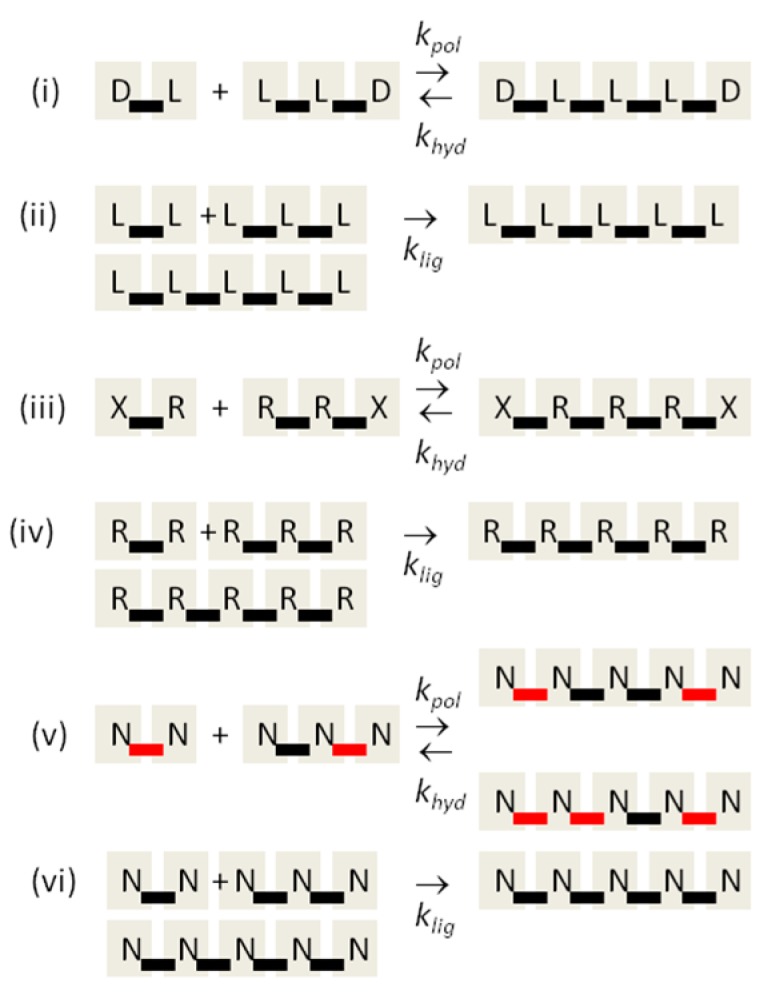
Reaction schemes for synthesis of RNA oligomers, involving uncatalyzed polymerization, *k_pol_*; hydrolysis, *k_hyd_*; and template-directed ligation, *k_lig_*. (**i**,**ii**) the chirality problem; (**iii**,**iv**) the monomer selection problem; (**v**,**vi**) the backbone regioselectivity problem.

The reaction schemes used in this paper are summarized in Figure 1. For the chirality problem, we begin with equal concentrations of monomers of the two enantiomers *D* and *L*. These monomers can react to form oligomers of all possible sequences. Uncatalyzed polymerization is controlled by a reaction rate constant *k_pol_*. Let *i* and *j* represent any two oligomers or single monomers, and let *ij* represent the longer oligomer formed by joining these two (see example in Figure 1i). Let *C_i_*, *C_j_,* and *C_ij_* be the concentrations of these three oligomers. The rate of formation of *ij* by uncatalyzed polymerization is *k_pol_C_i_C_j_*. The reverse hydrolysis reaction occurs at rate *k_hyd_C_ij_*. Hydrolysis of *ij* can also occur at any other point in the sequence. For simplicity, we suppose that the same rate constant *k_pol_* applies for joining any two oligomers and that the same rate constant *k_hyd_* applies for hydrolysis at any point in any sequence.

In addition to polymerization and hydrolysis, we suppose that uniform strands can act as templates for template-directed ligation of two shorter oligomers of the same kind. Thus, in Figure 1ii, an *LLLLL* pentamer can be a template for the ligation of *LL* and *LLL* to make another *LLLLL*. The same also applies for uniform *D* sequences. In order for the reaction to be template-directed, we suppose that both short oligomers *i* and *j* must be fully uniform (only *D* or only *L* this case) and that the template must contain a uniform sequence at least as long as the concatenated *ij* sequence. Thus, the sequence *DLLLLLLDDD* is also a template for joining *LL* and *LLL*. For simplicity, we suppose that all strands that possess a uniform sequence of the minimum required length are equally good templates. Template-directed ligation is controlled by rate constant *k_lig_*. The rate of ligation of *i* and *j* by templating is $k_{lig}C_{i}C_{j}C_{ij}^{temp}$, where $C_{ij}^{temp}$ is the total concentration of all strands that are templates for formation of *ij*, using the rules above. The templating reaction is treated as a single-step reaction. We do not keep track of duplex states and we assume that *ij* separates immediately from the template once formed. It would be possible to add separate steps of duplex formation and separation to the model, but initially we are aiming for the simplest possible model. 

A final feature of the chirality model is that it must be possible to convert monomers from one enantiomer to the other in some way. The model is initialized with equal concentrations of *L* and *D*. If no interconversion is possible, then no chiral symmetry breaking can occur. Direct racemization of sugars and nucleotides is extremely slow, but breakdown of a monomer to achiral precursors can also occur, as well as synthesis of monomers of both enantiomers from the precursors. We do not include precursors in this model. We assume that interconversion of *L* to *D* and *D* to *L* can occur by an effective single-step reaction at rates *k_int_C_L_* and *k_int_C_D_*, respectively. Only single monomers can be interconverted; monomers contained in oligomers cannot be changed until they are hydrolyzed back to single monomers. Including interconversion in this simple way means that the total concentration of nucleotides (including those in oligomers) is fixed, but the concentrations of *D* and *L* can each increase or decrease. 

For the monomer selection problem, we consider two monomers *R* and *X*, where *R* represents a ribonucleotide, and *X* represents an alternative nucleotide. The simplest case for the monomer selection problem is identical to the chirality problem, except that the labels *D* and *L* are replaced by *R* and *X* (see Figure 1iii,iv). We are assuming that formation of mixed *RX* sequences is possible by uncatalyzed polymerization, but that only uniform *R* or *X* sequences can be joined by templating. In the chirality case, there is complete symmetry initially between *D* and *L*; hence, the reaction rate constants should be equal for oligomers of *D* and *L*. This is not necessarily true for *R* and *X* because they are chemically different. Therefore, in the results section, we will consider cases in which one or other type of monomer is a better template than the other. For simplicity, we allow the interconversion between *R* and *X* to occur in a single effective step, although breakdown and re-synthesis of could be included in a more complex model. The interconversion rate constants in the two directions need not be equal in this case.

The backbone regioselectivity problem requires a few small modifications to the model. There is only one kind of monomer, which we denote *N* for nucleotide, and two kinds of bonds. We have coloured the bonds red and black in Figure 1v, representing 2′-5′ and 3′-5′ bonds, respectively. Joining two oligomers can occur via either type of bond formation, leading to two longer oligomers with different bond sequences. In the simplest case, we suppose the rate constants *k_pol_* and *k_hyd_* are the same for the two bond types, but this can be relaxed. The templating reaction occurs only if the two short oligomers are of uniform bond type, and only formation of the bond of the same type is catalyzed by the template (Figure 1vi). To be a template, the templating sequence must contain a uniform sequence of bonds at least as long as the sequence of bonds that is formed by the ligation reaction. Thus, in Figure 1vi, the pentamer formed has four black bonds, so the template must have at least four black bonds. There is no equivalent of the interconversion reaction in the regioselectivity problem because there is only one type of monomer. When a dimer with one kind of bond is hydrolyzed, the two monomers can be rejoined with either bond type, so the number of bonds of each type is not fixed. 

### 2.2. Computational Methods

In this paper, we study the three theoretical models described above using two kinds of computer simulations: reaction kinetics and Monte Carlo. In the reaction kinetics method, we keep track of the concentration of each monomer and oligomer species as a function of time, and solve the deterministic reaction kinetics equations by iterating forwards in small time steps of length *δt*. The change in concentration of oligomer *ij* due to formation from *i* and *j* and hydrolysis back to *i* and *j* is:

(1) $$\delta C_{ij}=\left(k_{pol}C_{i}C_{j}+k_{lig}C_{i}C_{j}C_{ij}^{temp}-k_{hyd}C_{ij}\right)\delta t$$

There is an equal and opposite change in concentration of *i* and *j* from these reactions:

(2) $$\delta C_{i}=\delta C_{j}=-\delta C_{ij}$$

We consider each possible pair *i* and *j* and sum up the total concentration changes of all molecules from all possible reactions. The concentration of the template in Equation (1) is different for each *i* and *j*. The template concentration does not change in the reaction for which it is a template, but templates are made by equivalent reactions involving other oligomers. Additionally, there is a change of the two monomer concentrations due to the interchange reaction described above:

(3) $$\delta C_{L}=-\delta C_{D}=k_{\mathrm{int}}(C_{D}-C_{L})\delta t$$

A slight modification is required for Equation (1) for the case of backbone regioselectivity. Whereas there is only one way to link two oligomers for the chirality and monomer selection problems, there are two ways to do this for the backbone regioselectivity problem. We therefore change the first term of Equation (1) so that each of the two reactions occurs at rate $\frac{1}{2}k_{pol}C_{i}C_{j}$. The total rate of linking *i* and *j* is still $k_{pol}C_{i}C_{j}$, which means that the distribution of lengths of oligomers is still the same as the other two models when there is no ligation term (See Appendix A). On the other hand, only one of the two kinds of bonds is catalyzed by the template-directed reaction. Therefore, the rate of this term remains a $k_{lig}C_{i}C_{j}C_{ij}^{temp}$, as with the other two models.

In the reaction kinetics method, we deal with all possible reactions deterministically; therefore, it is necessary to specify a maximum possible length, *l_max_*, of oligomers that can be formed, in order to keep the number of possible sequences finite. If *i* and *j* have a total length greater than *l_max_*, this polymeration reaction is not permitted to occur. For the results presented here, we set *l_max_* = 6. Thus the reaction system consists of 2^6^ hexamers, plus all the oligomers shorter than 6. We specified the total monomer concentration as *C_tot_* = 10 monomers per unit volume (arbitrary units). The rate constants for polymerization and hydrolysis were fixed at *k_pol_* = 1 per unit time per unit concentration and *k_hyd_* = 1 per unit time. The behaviour of the model was studied for different values of *k_lig._*

In the Monte Carlo method, we begin with a finite total number of nucleotides, *N_tot_*, and follow individual joining and hydrolysis reactions, keeping track of the sequences of all the oligomers that are formed. In this case, the computation is finite because of the finite number of molecules; therefore, it is not necessary to specify a maximum strand length. The oligomers are assumed to be reacting in a well-mixed solution of volume *V*. Reactions occur randomly with probabilities such that the expected rates are the same as the reaction kinetics method. We set *N_tot_* = 100,000 and *V* = 10,000; hence *C_tot_* = 10, as in the reaction kinetics method.

The Monte Carlo program also proceeds in small time steps of length *δt*. In each time step, each current strand is given a possibility of hydrolysis. For a strand of length *n* nucleotides, there are *n* − 1 bonds. Hydrolysis occurs with probability

(4) $$p_{hyd}=(n-1)k_{hyd}\delta t$$

and one of the *n* − 1 bonds is chosen at random.

Let *N_strands_* be the number of strands in the simulation (counting single monomers as a strand). *N_strands_* varies during the simulation and must be calculated at each time step, whereas the total number of nucleotides, *N_tot_*, is fixed. In each time step, each current strand is given a possibility to participate as the ‘left’ oligomer of a polymerization reaction (*i*). One of the *N_strands_* − 1 other strands is chosen to be the ‘right’ oligomer (*j*). The probability of the polymerization reaction occurring is

(5) $$p_{pol}=\frac{\left(N_{strands}-1\right)}{V}k_{pol}\delta t$$

If the reaction occurs, the new strand *ij* is formed by linking *i* and *j*.

The *i* + *j* reaction can also occur via a template-directed reaction. There are *N_strands_* − 2 other strands that could be the template. If all strands were templates, the probability of this reaction occurring would be:

(6) $$p_{lig}=\frac{\left(N_{strands}-1)(N_{strands}-2\right)}{V^{2}}k_{lig}\delta t$$

For each strand *i*, a second strand *j* is chosen at random, and a third strand *k* is chosen to be a potential template. If *k* is a template for the *i* + *j* reaction (as defined by the rules in Section 2.1), then the new strand *ij* is formed by the ligation reaction with probability *p_lig_.* If *k* is not a template, this reaction does not occur. At the beginning of the simulation, when all nucleotides are single monomers, *N_strands_*/*V* can be larger than 1. Also, we need to consider cases where *k_lig_* is much larger than 1 in order for the symmetry breaking to occur. Therefore, we begin with a very small *δt* (5 × 10^−5^) to ensure that *p_lig_* is a probability that is less than 1. As the simulation proceeds, the number of strands decreases, and it is possible to make *δt* larger, to increase the efficiency of the program. We adjust *δt* so that *p_lig_* remains close to 5%, or so that *p_pol_* is close to 5% in cases where *p_pol_* > *p_lig_*.

The interchange reaction is straightforward in the Monte Carlo simulation: each single monomer has a probability *k_int_δt* of switching from *D* to *L* or vice versa.

## 3. Results

### 3.1. Chirality

**Figure 2:**
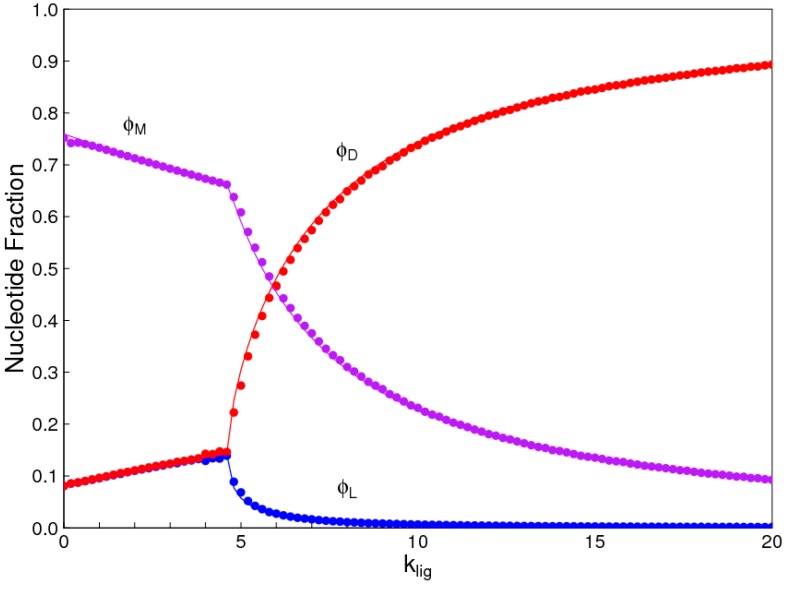
Fractions of nucleotides $\phi_{D}$, $\phi_{L}$, and $\phi_{M}$, in uniform *D* and *L* strands and in mixed strands. Smooth lines are calculated from reaction kinetics. Symbols are from Monte Carlo simulations. Below the phase transition, *D* and *L* are equal. Above the phase transition, there is a chiral bias in favour of *D*. An equivalent solution with the bias in the other direction is also possible.

The parameters in the results shown here are *C_tot_* = 10, *k_pol_* = 1, *k_hyd_* = 1, *k_int_* = 1, and *l_max_* = 6, as described above. Figure 2 shows the faction of nucleotides $\phi_{D}$, $\phi_{L}$, and $\phi_{M}$ at equilibrium. The fraction of nucleotides contained in uniform *D* oligomers is

(7) $$\phi_{D}=\frac{1}{C_{tot}}\sum_{i}n_{i}C_{i}$$

where *n_i_* is the number of nucleotides in sequence *i*, *C_i_* is the concentration of sequence *i*, and the sum is over all sequences that are uniform-*D* oligomers (dimers and longer). Similarly, we can define the fraction of nucleotides in uniform-*L* oligomers, $\phi_{L}$, and in mixed oligomers, $\phi_{M}$. In the latter case, the sum is over all sequences that contain at least one *D* and one *L*.

When *k_lig_* = 0, a majority of nucleotides end up in mixed oligomers. The fraction of nucleotides in uniform *D* and *L* oligomers are both low, and equal to one another (Figure 2). There is a symmetry-breaking phase transition close to *k_lig_* = 4.6 in this example. Above this point, the fraction of monomers in one kind of uniform oligomers is much higher than in the other kind (in this case $\phi_{D}$ > $\phi_{L}$), and the fraction in mixed oligomers also decreases rapidly.

Symmetry breaking occurs because the symmetric mixture is unstable when *k_lig_* is large. The simulations demonstrate this by beginning with a small bias towards *D* monomers—*C_D_* = 0.505*C_tot_* and *C_L_* = 0.495*C_tot_*. When *k_lig_* is low, the bias disappears, and the equilibrium solution is perfectly symmetric. When *k_lig_* is high, the bias increases, and the equilibrium has a large excess of *D* over *L*. There is an equivalent equal-and-opposite solution with an excess of *L* over *D*, which is reached if we began with a slight bias towards *L*. In any real case, there is a finite volume with a finite number of molecules, and small fluctuations are bound to create a slight asymmetry one way or the other. This asymmetry is magnified to a large excess of either *L* or *D* because the reaction system is inherently unstable. The prediction is that the system will become biased towards *L* or *D* with equal probability if it begins with equal concentrations of the two. It is not necessary to begin with any source of initial asymmetry. On the other hand, if the reaction system begins with a slight bias one way for other reasons (as is apparently observed for organic molecules in meteorites [30]), the autocatalytic reaction amplifies the asymmetry in the direction that is previously specified (theoretical examples of this are given in [10]).

As a further check that our simulations have reached an equilibrium, we also started simulations where *C_D_* = *C_tot_* and *C_L_* = 0. These simulations were run for a long time with no interchange reaction permitted, so that a distribution of uniform *D* strands was created. The interchange reaction was then turned on, and the simulation was continued to equilibrium. For low *k_lig_*, the same symmetric solution was reached as before, and for high *k_lig_,* the same chirally biased solution was reached as before. This shows that the biased solution can be reached either by amplifying a very small initial bias, or by allowing a system with a 100% chiral bias to relax to the equilibrium state.

We also used the Monte Carlo simulation to study the same case. The points in Figure 2 are from Monte Carlo, and the lines are from the reaction kinetics. These two methods give identical results, which is an important check on validity of both methods. In the Monte Carlo case, we calculate time averages of quantities once equilibrium has been reached. We can begin with exactly equal numbers of *D* and *L* monomers (*N_tot_*/2 = 50,000 of each type). It is not necessary to begin with a small bias in one direction, because the interchange reactions allow fluctuations of these numbers to arise. If *k_lig_* is above the phase transition, the fluctuations are amplified and the system shifts to a state with either high *D* or high *L* with equal probability. For the purposes of comparison with the reaction kinetics method in Figure 2, we plotted the higher concentration as *D* and the lower one as *L* in each case, but the labeling of *D* and *L* is arbitrary because there is a true symmetry in this problem and the symmetry breaking can go either way.

**Figure 3:**
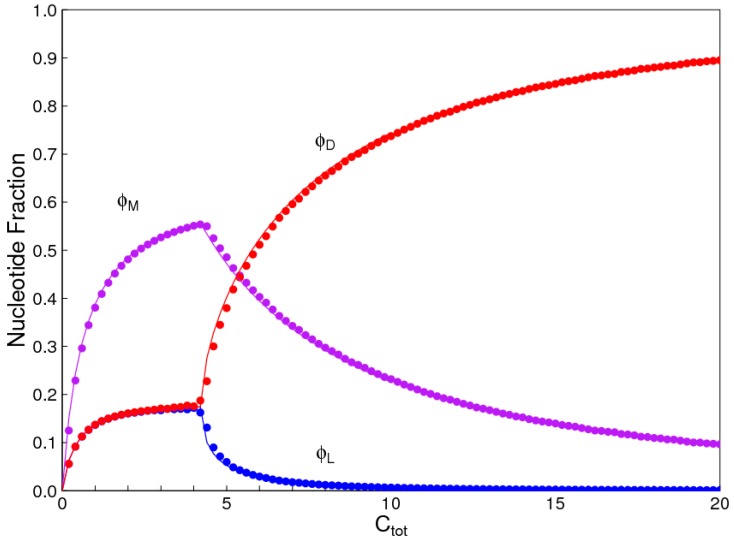
Fraction of nucleotides $\phi_{D}$, $\phi_{L}$, and $\phi_{M}$, in uniform *D* and *L* strands and in mixed strands as a function of concentration. Smooth lines are calculated from reaction kinetics. Symbols are from Monte Carlo simulations.

The reactions for hydrolysis, polymerization, and template-directed ligation are unimolecular, bimolecular, and trimolecular, respectively. Thus, the relative rates of these reactions depend on the concentration. The importance of templating is increased when the concentration is high. Figure 3 shows the nuclotide fractions as a function of total monomer concentration, *C_tot_*, with *k_lig_* = 10, *k_pol_* = 1, *k_hyd_* = 1, and *k_int_* = 1. At low concentration, *D* and *L* are equal, and chiral symmetry breaking occurs as *C_tot_* is increased. If this is repeated for different *k_lig_* rates, the concentration at which the symmetry breaking occurs is roughly inversely proportional to *k_lig_*.

**Figure 4:**
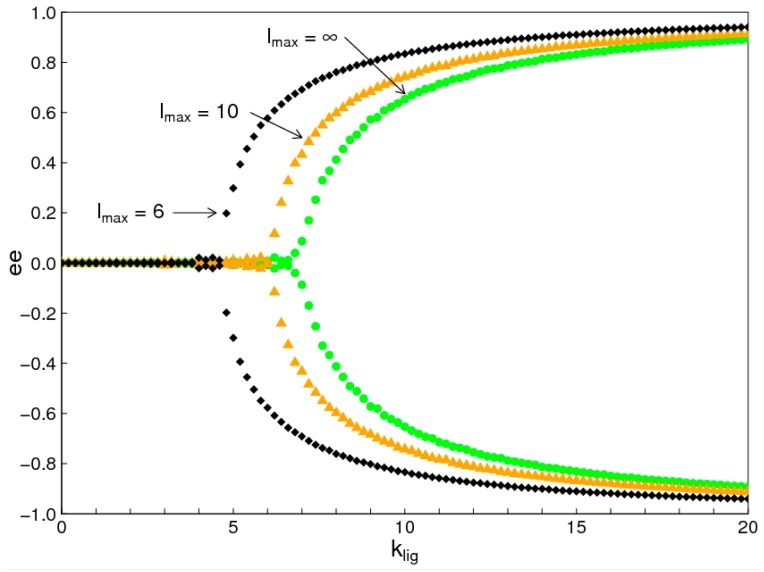
Enantiomeric excess (*ee*) of nucleotides contained in oligomers. The cases of *l_max_* = 6, 10, and ∞ are very similar, but the chiral symmetry breaking occurs at slightly higher *k_lig_* when *l_max_* is larger. The *ee* is zero below the phase transition, and can either be positive or negative once the symmetry is broken.

Figure 2 and Figure 3 were done with maximum strand length *l_max_* = 6, in order to allow comparison of results between the two simulation methods. Using the Monte Carlo program, we repeated the simulation with *l_max_* = 10, and with no maximum length restriction (*l_max_* = *∞*). The results are similar to the case with *l_max_* = 6, although the phase transition occurs at a higher value of *k_lig_*. This is shown in Figure 4 in terms of enantiomeric excess, *ee*. The *ee* is defined as the difference in concentrations of the two enantiomers relative to the sum: ee=(D−L)/(D+L). Figure 4 shows the *ee* for nucleotides incorporated into oligomers (length 2 or above). There are two equal and opposite solutions when *k_lig_* is high. In our model, there is direct interchange between single monomers of *D* and *L*. Therefore, the concentrations of the two single monomers remain equal. For this reason, the *ee* of single monomers is always zero, even above the phase transition, whereas the *ee* of the nucleotides in oligomers becomes non-zero above the phase transition. The main point of Figure 4 is that the bifurcation point shifts to slightly higher *k_lig_* as the *l_max_* is shifted from 6 to 10, to ∞, but there is no qualitative change. Imposing the maximum length restriction simplifies the simulations slightly, but states of biased chirality arise whether or not there is a maximum length. The equilibrium distribution of lengths of oligomers for these cases is discussed in the Appendix of this paper.

**Figure 5:**
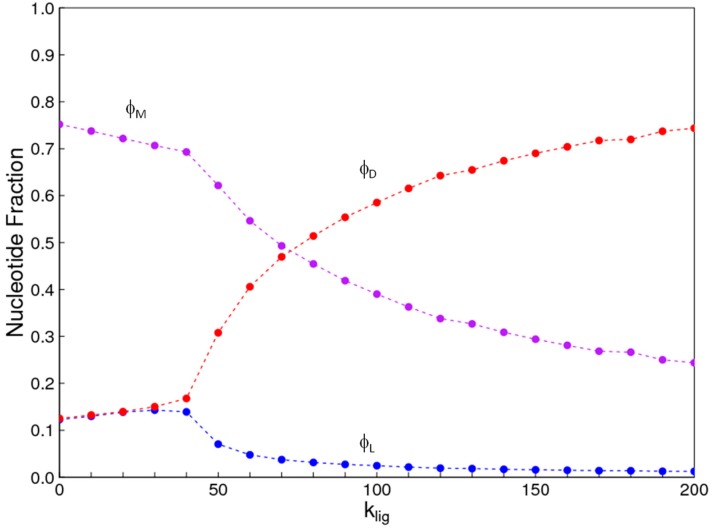
Fractions of nucleotides $\phi_{D}$, $\phi_{L}$, and $\phi_{M}$, in uniform *D* and *L* strands and in mixed strands for the case where A, C, G, and U bases of both enantiomers are explicitly included and complementary pairing is enforced between templates and oligomers being ligated.

For the results shown above, we considered *D* and *L* nucleotides without specifying the base sequence. In order to deal more explicitly with complementary sequence pairing, we also considered a case in which there are eight kinds of nucleotides: A, U, G, and C bases, each of *D* and *L* form. Polymerization and hydrolysis reactions occurred at equal rates independently of the base and the chirality. For template-directed reactions to occur, both the oligomers *i* and *j* had to be of uniform chirality, and the same chirality as each other, but they could have any base sequence. A third sequence could be a template if it contained a uniform sequence of the same chirality as *i* and *j,* with a base sequence that was complementary to the *ij* sequence that is formed by ligation. Only AU and GC pairs were permitted when checking for complementarity. The results of this case, shown in Figure 5, are very similar to Figure 2, except that the phase transition occurs at *k_lig_* close to 40 rather than 4.6 in Figure 2. The main effect of introducing the detailed requirement of complementary pairing is to reduce the concentration of strands that can be catalysts for any two oligomers being ligated. As a result, higher values of *k_lig_* are required in order for the symmetry-breaking effects of the template-directed reaction to become apparent. However, the essential symmetry-breaking effect is the same as in the simpler model.

### 3.2. Monomer Selection

**Figure 6:**
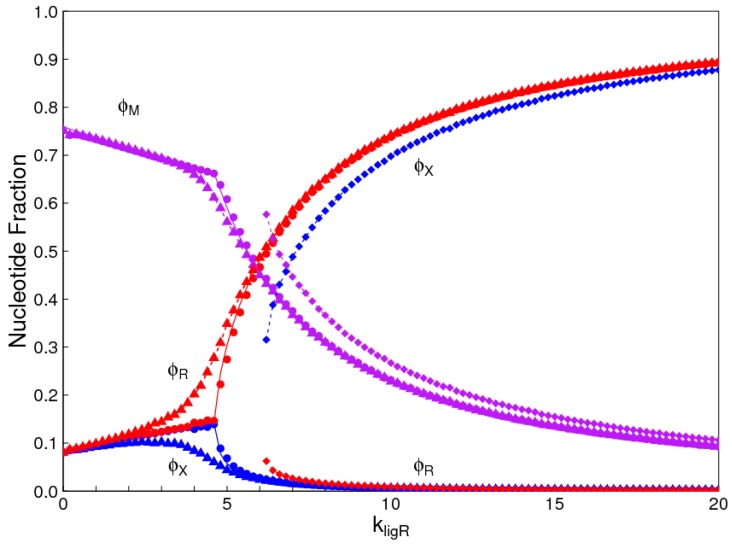
Comparison of the monomer selection problem with the chirality problem. Red points: uniform *R* strands; Blue points: uniform *X* strands; Violet points: mixed strands. Circles show the solution in the case where there is perfect symmetry between *R* and *X* (This is identical to Figure 2). Triangles and squares show the two possible solutions in the case where $k_{ligX}=0.9k_{ligR}$.

In this problem, *R* represents a ribonucleotide and *X* represents an alternative nucleotide. If the rate constants for the two nucleotides are the same, the problem is identical to the chirality problem. The symmetry between *R* and *X* is broken as *k_lig_* is increased. In Figure 6, we also considered the case where the ligation rates for the two types of polymer are slightly different: $k_{ligX}=0.9k_{ligR}$. This means that *R* has an advantage over *X* because the ligation rate of uniform *X* sequences is less than that for uniform *R* sequences. It can be seen that the phase transition is ‘rounded out’ when there is no exact symmetry between *R* and *X*. When *k_lig_* is far above or far below the phase transition, the concentrations of *R* and *X* are close to that for the symmetric case. 

Figure 6 also shows a second solution at high *k_ligR_* where the symmetry is broken in the opposite direction, i.e., *X* dominates even though it has a lower ligation rate than *R*. This solution only exists above a minimum ligation rate (close to 6 in the figure), whereas the solution where *R* dominates exists for all values of *k_ligR_*. The two solutions are obtained by beginning with monomers of all *R* or all *X* and allowing a steady state to be reached before the interchange reaction is turned on. The interchange reaction is then turned on and the simulation is then continued to equilibrium. For ligation rates where there is only one solution, the same solution is reached starting from both extremes. When there are two solutions, these are reached from the two different starting points. It should be remembered that, even in the simplest case in Figure 2, there are two solutions when *k_lig_* is above the transition point. As these are equal and opposite, only one set of curves appears in Figure 2, but the two opposite solutions are visible in the *ee* graph (Figure 4). In Figure 6, the two solutions are not equal and opposite; therefore, two sets of curves are visible.

A second way in which *R* and *X* can differ is by their frequency in the monomer mixture prior to polymerization. If the rate constants for interchange from *X* to *R* and from *R* to *X* are *k_intXR_* and *k_intRX_*, respectively, then the frequencies of monomers at equilibrium under the interchange reaction, in absence of any polymerization will be unequal:

(8) $$\frac{C_{X}}{C_{R}}=\frac{k_{intRX}}{k_{intXR}}$$

**Figure 7:**
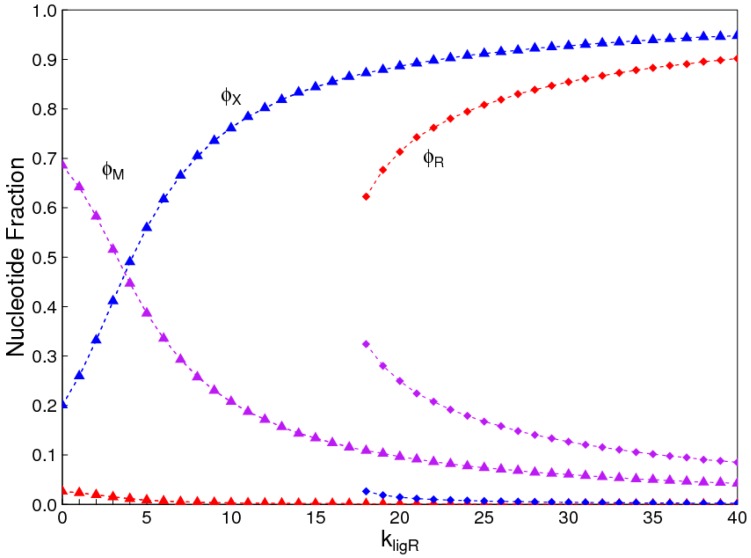
Two possible solutions in the monomer selection problem where $C_{X}=2C_{R}$ and $k_{ligX}=0.5k_{ligR}$. Red points: uniform *R* strands; Blue points: uniform *X* strands; Violet points: mixed strands. Triangles: solution where *X* dominates; Squares: solution where *R* dominates.

Figure 7 shows the case where there is a two-fold advantage to *X* in terms of equilibrium monomer frequencies ($C_{R}=\frac{1}{2}C_{X}=\frac{1}{3}C_{tot}$), but a two-fold advantage to *R* in terms of ligation rates $k_{ligX}=0.5k_{ligR}$. In this case, the solution where *X* dominates exists over the whole range of ligation rates, and a second solution where *R* dominates exists only for high ligation rates. Thus, the frequency of the monomers in the reaction mixture is a major factor that determines which type of monomer dominates when templating occurs. 

**Figure 8:**
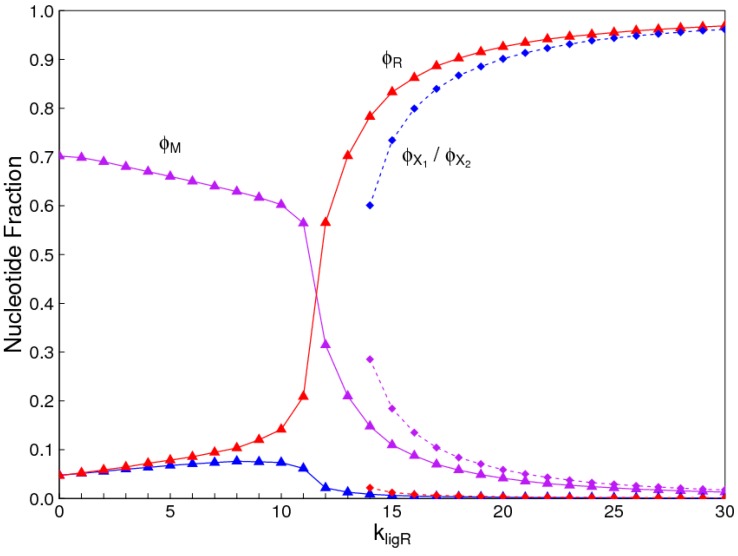
Possible solutions in the case where there are three kinds of nucleotides, *R*, *X*_1_, and *X*_2_, with equal monomer frequency and $k_{ligX_{1}}=k_{ligX_{2}}=0.9k_{ligR}$. Triangles: solution where *R* dominates; Squares: two equivalent solutions where either *X*_1_ or *X*_2_ dominates.

The number of monomer types in the mixture need not be limited to only two. In Figure 8, we consider the case where there are two alternatives, *X*_1_ and *X*_2_, in addition to *R*. We assume that all the interchange rates are equal, so all three types have equal average concentration in the monomer mixture ($C_{R}=C_{X_{1}}=C_{X_{2}}=\frac{1}{3}C_{tot}$), and we give *R* a small advantage in the ligation rate ($k_{ligX_{1}}=k_{ligX_{2}}=0.9k_{ligR}$). In this case, it is the solution where *R* dominates that is possible over the full range of ligation rates, and the two solutions where the other monomer types dominate exist only at high ligation rates.

### 3.3. Regioselectivity

**Figure 9:**
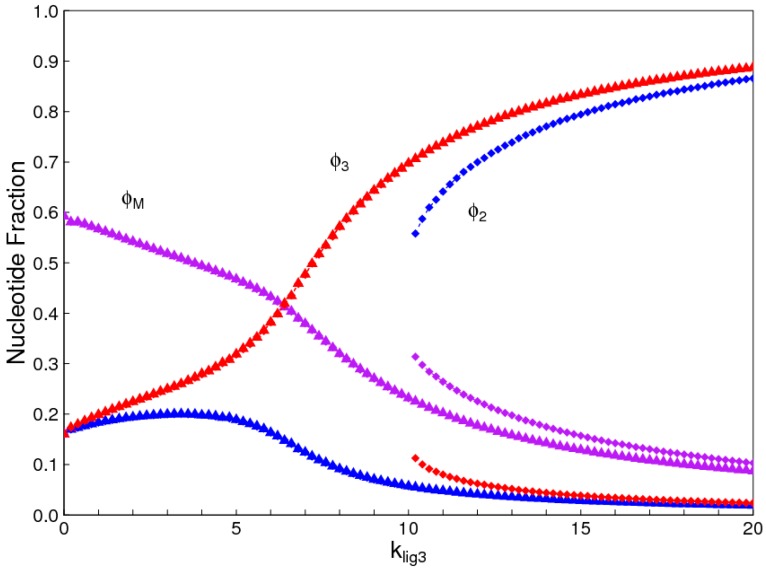
Concentration of nucleotides in uniform 3′-5′ and 2′-5′ strands and in mixed strands for the case where $k_{lig2}=0.9k_{lig3}$. Red points: uniform 3′-5′ strands; Blue points: uniform 2′-5′ strands; Violet points: mixed strands. Triangles show the solution where 3′-5′ strands dominate and squares show the solution where 2′-5′ strands dominate.

If we assume a complete symmetry between 3′-5′ bonds and 2′-5′ bonds, then the regioselectivity model behaves similarly to the chirality model. There is a symmetry-breaking phase transition qualitatively similar to Figure 2, in which one or other of the two bond types dominates when *k_lig_* is high. We do not show this case, because there are many ways in which these two bond types might differ in practice, and there is no reason to suppose perfect symmetry. In Figure 9, we suppose the major difference in the two bond types is that the uniform 3′-5′ oligomers have a higher ligation rate than the uniform 2′-5′ oligomers ($k_{lig2}=0.9k_{lig3}$). It is seen that there is a solution where 3′-5′ strands dominate that is possible over the full range of ligation rates, and a solution where 2′-5′ strands dominate that is possible only at high ligation rates. The other rate parameters, *k_pol_* and *k_hyd_*, are assumed to be equal in this example, but are likely to be different in an experimental system. Which of the bond types dominates will be a function of the rates of all these parameters for both bond types.

## 4. Discussion

In the introduction, we reviewed the experimental evidence that oligomers with uniform chirality, monomer composition, or bond type are effective templates for the growth of complementary strands with the same chirality, monomer composition, or bond type, and that oligomers that are mixed in any of these properties are less effective templates than uniform oligomers. The computational models studied in this paper incorporate these features, and show that, when template-directed ligation is fast compared to random polymerization without a template, we expect that uniform biopolymers will emerge, and that they are homochiral, use a restricted monomer set, and have a highly regioselective backbone. We thus argue that these essential properties of biological nucleic acids have emerged as a result of the importance of template-directed reactions in the early stages of evolution. Although models for the emergence of homochirality have been widely studied previously, the link between chirality, monomer selection, and regioselectivity has not been emphasized in the past. The present theory therefore contributes by showing in a simple way why these three problems are very similar. These results lead us to expect that RNA with the three uniform properties seen in biology could emerge from a prebiotic chemical mixture *before* the origin of life, in the sense that the proposed mechanism requires only the existence of short oligomers that can undergo template-directed replication, but it does not require the existence of specific sequences that would act as catalysts (ribozymes). 

The mechanism we discuss here depends on the ability of nucleic acids to be templates; hence, it applies to RNA and polymers with similar structures, but not to other kinds of biomolecules that are also chiral in today’s organisms but cannot be templates. The ability of nucleic acids to be templates is, of course, a prime argument in favour of some kind of RNA World scenario for the origin of life. In an origins scenario where RNA replication arises early, and where ribozymes are the initial biological catalysts, it is possible to explain the transfer of chirality from RNA to other biomolecules by the fact that chiral RNAs catalyze the synthesis of the other molecules. In particular, if protein synthesis depended on chiral tRNAs and rRNAs, then amino acids of the appropriate chirality could be charged onto tRNAs, and protein sequences of uniform chirality could be synthesized. This would be true, even if the mixture of single amino acids were completely racemic, because amino acids of the wrong enantiomer would not be recognized by the ribozymes. On the other hand, if the amino acids were already chiral for some other reason at the time of the invention of protein synthesis, the early ribosome would simply make use of the existing supply of chiral amino acids. 

Related to this is the possibility that the transfer of chirality was in the other direction: from amino acids to sugars and nucleotides. Several studies have shown [31,32] that chiral amino acids can catalyze the synthesis of chiral glyceraldehye and other sugars. There is also evidence for a moderate enantiomeric excess in amino acids found in meteorites [30], which is presumed to originate by an abiotic mechanism outside the Earth, such as chirally biased degradation under the influence of circularly polarized light. We have previously considered the way that a small extraterrestial chiral bias can contribute to scenarios involving the emergence of homochirality [10]. Understanding the direction of chiral transfer will require a more detailed picture of the full network of reactions involving biomolecules of all kinds at the time of the origin of life. The template-based mechanism presented here is not in conflict with other studies showing transfer of chirality from one molecule to another, whichever the direction of transfer, and it is quite possible that more than one mechanism jointly contributed to the creation of a biology in which several kinds of chiral biopolymer are inter-dependent. Nevertheless, we maintain that templating must have been an essential part of this process. Life requires the copying of sequence information that occurs via the template-directed synthesis of complementary strands of nucleic acids. A mixture of small molecules (whether chirally biased or not) that did not possess a mechanism of sequence replication would not yet constitute life, in our view. Templating is a straightforward mechanism that ‘purifies’ biopolymers at the level of chirality, chemical composition, and regioselectivity, as we have shown. The point of this paper is to show that templating alone can provide a mechanism for the emergence of all these properties, even in absence of other sources of asymmetry. If other sources, or other mechanisms, exist, then templating magnifies these effects to a much greater extent.

The mechanism for the emergence of RNA discussed in the present paper is an example of what we have termed *chemical evolution* [33]. The main distinctions between chemical evolution and biological evolution, as defined in [33], are (1) that, for chemical evolution, the major part of the sequence diversity on which natural selection acts is provided by random polymer synthesis, rather than by mutations occurring during replication of previously existing sequences; and (2) that selection is acting on physicochemical properties possessed by all short oligomers (like ligation and hydrolysis rates) rather than on encoded function of long sequences (such as ribozymes) that are only possessed by a small number of sequences in a large sequence space. If RNA can emerge directly from a prebiotic mixture by chemical evolution, as envisaged here and previously [15,16], then it is not necessary to consider more complex pathways requiring the evolution of biological function in some pre-RNA polymer and the subsequent transfer of sequences and function to RNA, as has sometimes been proposed [34].

The monomer selection problem is probably the most complex and least understood of the three problems considered in this paper. It is clear that the real world is much more complicated than the simple model we have studied. For example, hybrid duplexes of RNA, DNA, and TNA were studied experimentally [17], and it was shown that the details of the nucleotide sequences and backbone structures significantly affect the melting temperatures (T_m_) of helices, and presumably also affect the rates of polymerization, hydrolysis, and ligation. Table 2 of [17] illustrates how complex this problem is. There are 14 cases where the hybrid duplex has a lower T_m_ than the uniform duplexes of both polymers, 8 cases where the hybrid has a T_m_ in between the two uniform duplexes, and 2 cases where the hybrid has a T_m_ higher than both the uniform duplexes. The latter two cases are surprising, but the effect only works one way round: if the sequences are switched, the hybrid has a lower T_m_ than both uniform duplexes. Although the thermodynamic details are rather complex, there is a general tendency to favour uniform duplexes, which is the essential point that we assume in our model. The case where both uniform duplexes are good templates and the hybrids are weak corresponds to the case studied here. There is an approximate symmetry between the two uniform polymers that is broken when the template-directed reaction is fast. If the hybrid is intermediate between the two uniform duplexes, then there will be a straightforward selection for the better of the two uniform systems. The monomer selection problem does not require symmetry breaking in that case. 

We hope to continue this work to consider important details not included in the present paper, including differences in thermodynamic properties and rate constants between different base sequences with the same backbone, and adding separate steps for helix formation and melting, rather than combining them into a single effective ligation step. The simple model presented here is sufficient to demonstrate the similarity between the problems of chirality, monomer selection, and regioselectivy, and to establish the relevance of symmetry breaking transitions in all three cases.

## Figures and Tables

**Figure A1:**
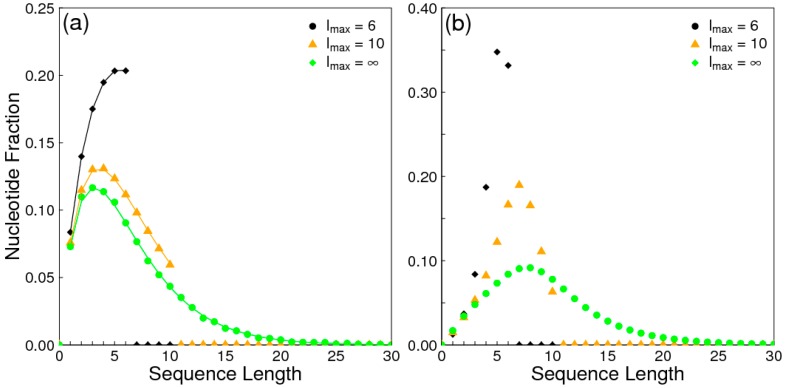
Fraction of nucleotides, $f_{n}$, in strands of length *n*. (**a**) No templating, *k_lig_* = 0. Points = simulation. Lines = analytical calculation from Equations (A2) to (A4). (**b**) High templating rate, *k_lig_* = 20. Points = simulations.

## Appendix A

Here we consider the distribution of lengths of oligomers and the effect of template-directed reactions on this distribution. In most models of polymerization in which rates of linking and breaking monomers are independent on the lengths of the oligomers, e.g., [35], the equilibrium distribution of lengths is exponential, and it is often simple enough to be calculated exactly. If there is no templating (*k_lig_* = 0), the model in this paper is almost the same as that discussed in [35], and the length distribution can be calculated in the same way. Let *C_n_* be the total concentration of oligomers of length *n*. At equilibrium, the rates of polymerization and hydrolysis must balance for each pair of lengths *n* and *m*:

(A1) $$k_{pol}C_{n}C_{m}=k_{hyd}C_{n+m}$$

This means that the solution is of the form

(A2) $$C_{n}=C_{1}{\left(KC_{1}\right)}^{n-1}$$

where $C_{1}$ is the equilibrium concentration of single monomers, and $K=k_{pol}/k_{hyd}$. The total nucleotide concentration *C_tot_* is fixed; hence

(A3) $$\sum_{n=1}^{l\max}nC_{n}=C_{tot}$$

Substituting (A2) into (A3), we obtain the following equation, from which the solution for $C_{1}$ can be found numerically.

(A4) $$\frac{C_{1}\left(1+l_{\max}{\left(KC_{1}\right)}^{l\max+1}-(l_{\max}+1){\left(KC_{1}\right)}^{l\max}\right)}{{\left(1-KC_{1}\right)}^{2}}=C_{tot}$$

To illustrate the shape of the strand length distribution, it is useful to plot the fraction of nucleotides in strands of length *n*—this is $f_{n}=nC_{n}/C_{tot}$. Figure A1a shows the theoretical curve calculated from Equations (A2) to (A4), together with simulation results. The fact that these are equal confirms that our simulation results are accurate in the case we can calculate analytically. Figure A1b shows the length distribution for *k_lig_* = 20, which is the largest rate considered in Figure 4. The addition of templating shifts the length distribution towards longer lengths. However, the length distribution always decays exponentially for long lengths, even when *k_lig_* is high and when *l_max_* = *∞.* The shapes of the length distributions in similar models to ours that include templating have also been considered by other authors [36,37].
